# Efficient implementation of the Hodgkin-Huxley potassium channel via a single volatile memristor

**DOI:** 10.3389/fnins.2025.1569397

**Published:** 2025-07-18

**Authors:** Lennart P. L. Landsmeer, Erbing Hua, Heba Abunahla, Muhammad Ali Siddiqi, Ryoichi Ishihara, Chris I. De Zeeuw, Said Hamdioui, Christos Strydis

**Affiliations:** ^1^Department of Quantum and Computing Engineering, Delft University of Technology, Delft, Netherlands; ^2^Neurocomputing Lab, Department of Neuroscience, Erasmus Medical Center, Rotterdam, Netherlands; ^3^Department of Electrical Engineering, Lahore University of Management Sciences, Lahore, Pakistan; ^4^Netherlands Institute for Neuroscience, Royal Academy of Sciences, Amsterdam, Netherlands

**Keywords:** realistic brain models, memristors, brain machine interfacing, neural networks, simulations

## Abstract

**Introduction:**

In 2012, potassium and sodium ion channels in Hodgkin-Huxley-based brain models were shown to exhibit memristive behavior. This positioned memristors as strong candidates for implementing biologically accurate artificial neurons. Memristor-based brain simulations offer advantages in energy efficiency, scalability, and compactness, benefiting fields such as soft robotics, embedded systems, and neuroprosthetics.

**Methods:**

Previous approaches used current-controlled Mott memristors, which poorly matched the voltage-controlled nature of ion channels. This study employs volatile, oxide-based memristors that leverage electric-field-driven oxygen-vacancy migration to emulate voltage-dependent channel behavior. We selected candidate WOx and NbOx memristors and modeled their dynamics to verify performance as Hodgkin-Huxley potassium channels.

**Results:**

The device exhibits sigmoidal gating and voltage-dependent time constants consistent with the theoretical model. By scaling the passive circuitry around the memristors, we show that they capture the essential mechanisms of potassium ion-channels, although spike height is reduced due to strong non-linear voltage-dependence. Still, by cascading multiple compartments, typical spike propagation is retained.

**Discussion:**

This is the first demonstration of a voltage-controlled memristor replicating the Hodgkin-Huxley potassium channel, validating its potential for more efficient brain simulation hardware.

## 1 Introduction

Neuroscientific research requires efficient and accurate simulations of the brain (Einevoll et al., 2019; Colombo, 2017; De Garis et al., 2010; Yamazaki et al., 2021). Efficiency entails low power consumption, minimal physical size and performance approaching biological speeds. Accuracy, on the other hand, requires fidelity to biological processes, specifically at the biophysical level. This is widely recognized as adherence to the well-validated Hodgkin-Huxley (*abbrv*. HH) model and its extensions, which simulate voltage-gated ion channels like those of sodium and potassium (Catterall et al., 2012; Hodgkin and Huxley, 1952). Consequently, there is a critical need for solutions that enable such simulations with both efficiency and precision.

**Figure 1:**
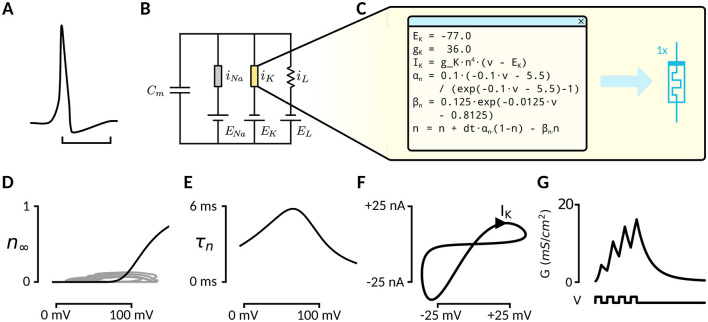
**(A)** The Hodgkin-Huxley model explains action potentials in neurons from the interplay between the transmembrane flux of sodium and potassium ions. The hyperpolarization phase is the main effect of a flux through the potassium ion channel. **(B)** Study setup: Currently, digital simulation is required for accurate simulation of the HH model. Memristors offer a much more efficient alternative but are not accurate yet. **(C)** Parameters and formulae describing potassium ion channel dynamics. **(D)** Steady-state gate behavior of the potassium ion channel. **(E)** Time-constant of the potassium ion gate. **(F)** A sinusoidal input voltage over the isolated potassium channel will evoke the typical hysteresis loop associated with memristor devices. **(G)** A pulsed input will lead to gradual increase in channel conductance, while absence of bias will lead to a decay into the high-resistance state, showing the volatile, voltage controlled nature of the channel.

The HH model stands as the standard model for accurate brain simulations, replicating neural spiking at the biophysical level (Figure 1A). At a high level, it is described as a circuit model with two ion channels (Figure 1B). The potassium channel operates in line with equations that capture its slow-moving nature (Figures 1D, E), while the sodium channel is modeled with equations that reflect both a slow-moving component and a fast negative differential resistance behavior. These components are key to replicating the dynamics of biological neurons. However, no classical electrical device accurately matches these biophysical ion-channel behaviors, resulting in inherently inefficient implementations in existing hardware.

Existing implementations of the HH model rely on digital, analog and memristor-based approaches, each with specific limitations. Digital implementations are inefficient due to the overhead of numerical discretization (Abi Akar et al., 2019; Carnevale and Hines, 2006; Panagiotou et al., 2022; Miedema et al., 2020; Landsmeer et al., 2024), while analog (CMOS) designs suffer from large component-count and die-area requirements (Alvado et al., 2004). Existing physical Mott-memristor based solutions (Pickett et al., 2013; Yi et al., 2018; Yang et al., 2024), though efficient, currently lack accuracy and do not represent HH models at the equation level (Lim et al., 2015; Nabil et al., 2022; Landsmeer et al., 2025). In a memristor-based solution, one memristor should represent one ion channel. Due to this one-to-one representation, memristors are the only type of solution that offers a high level of efficiency, setting them apart from digital- and analog-based solutions (Chua et al., 2012; Chua, 2013; Sah et al., 2014). As such, there is a need for memristor-based, ion channel replacement that is both efficient and accurate.

Memristors—as characterized by Chua's seminal insight (Chua et al., 2012; Sah et al., 2014)—are inherently aligned with the behavior of ion channels, suggesting that ion channels are, in fact, memristors. This is exemplified by the typical pinched-hysteresis loop that these channels exhibit under oscillatory bias (Figure 1F). To accurately represent HH ion channels, memristors must exhibit specific characteristics: volatility, analog behavior, voltage control and first-order dynamics. Additionally, they must capture the approximate time constants and dynamics unique to each channel. Despite this conceptual alignment, existing approaches to memristive HH implementations fall short either due to device limitations or an inability to replicate the nuanced biophysical properties of ion channels. These challenges underscore the need for refined memristor models tailored to this purpose.

**Figure 2:**
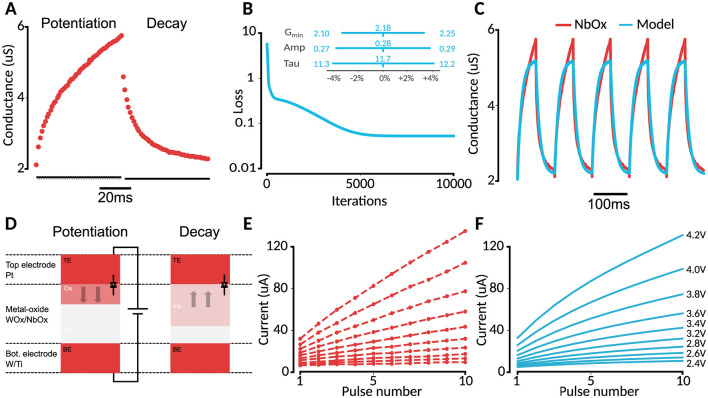
**(A)** NbOx experimental data (Ju and Kim, 2024). **(B)** Training loss during fitting and parameter sensitivity range for 10% MSE decrease. **(C)** Data vs model conductance. **(D)** Memristor model as explained by Lin et al. (2023) and Ju and Kim (2024). **(E)** Voltage-pulse experiment from Ju and Kim (2024). **(F)** Model reproduction.

We identified two candidate memristors for modeling the potassium channel, a tungsten oxide (WOx, Du et al., 2017) and niobium oxide (NbOx, Ju and Kim, 2024) memristor, by searching literature for existing devices showing the same pulse response as the potassium ion channel (Figure 1G). These materials exhibit properties that align with the requirements for HH ion-channel simulations, such as volatility and voltage-controlled behavior. This is most visible by comparing the pulse-response plot in Figure 1G with the potentiation-decay plot (Figure 2A); applying bias makes the device gradually more conductive, while the absence of external potential allows for a decay in conductance. While promising, these devices have no systematic evaluation within the specific context of HH channels. Such an evaluation is crucial for candidate memristors to determine their suitability and for advancing their application in accurate, efficient neural simulations.

The evaluation of such oxygen-diffusion dynamics in memristors for HH channels involves simulating the underlying physics of these devices. This approach builds on models such as those proposed by Du et al. (2015, 2017), which explain memristor behavior from interface-mediated Schottky effects. In our simulations, we allow for scaling in time, current and voltage, as these adjustments correspond to scaling of the remaining passive elements.

The potassium ion-channel can be modeled as a first-order, volatile, voltage-controlled memristor. While oxygen-vacancy-migration memristors have been used for simulating reservoir computing or synaptic functions, they were not demonstrated to correspond to the potassium channel or exhibit any direct relation to neuronal behavior (Du et al., 2017). The authors of that work propose a first-order system to capture memristor dynamics, incorporating contributions from both Schottky and tunneling mechanisms. They define the current *i* as the result of a modulated Schottky-barrier and tunneling at the insulator-electrode interface. The state variable *w* captures oxygen migration due to voltage biasing and decay over time. *w* is constrained between *w*_*min*_ and *w*_*max*_ via the window function *W*(*w*). The state equations describing this implementation are shown next:


(1) $$\begin{array}{ll} i= & \left(1-w\right)\alpha \left[1-e^{-\beta V}\right]+w\gamma \sinh\left(\delta V\right) \end{array}$$

(2) $$\dot{w}=W\left(w\right)\lambda \sinh\left(\eta V\right)-\frac{w-w_{min}}{\tau }$$

(3) $$\begin{array}{l} \;w\in \left[w_{min},w_{max}\right] \end{array}$$

(4) $$\begin{array}{ll} W\left(w\right)= & 1-\frac{\exp\left(w\right)}{\exp\left(3\right)} \end{array}$$

In this work, we aim to provide an efficient and accurate memristive implementation of the HH potassium ion channel (Figure 1C). As such, we make the following contributions:

- Identify WOx and NbOx devices as potential candidates for potassium-channel emulation.
- Model the memristor behavior of NbOx using the oxygen-migration model due to Du et al. (2017).
- Show that the modeled devices can replace the potassium channel in the Hodgkin-Huxley model, with both high efficiency and accuracy.

This manuscript is organized as follows: Section 2 details the materials and methods, including how the memristor model is parameterized given the available data, the tuning of scaling parameters to emulate the HH potassium channel, and the software and hardware used. Section 3 presents results, covering theoretical grounds for using the memristor model for potassium channel emulation, parameter tuning for the NbOx-memristor model, HH model scaling, system simulations, and energy usage, while also assessing WOx's suitability. Section 4 discusses memristors for HH emulation, comparisons with Mott-insulator neurons, potential future designs, and model validity.

## 2 Materials and methods

### 2.1 Memristor model

In contrast to the WOx model of Du et al. (2015), the selected NbOx candidate memristor lacks an established model for its behavior, but is known to operate via a similar oxygen-vacancy migration mechanism. Ju and Kim (2024) subjected the memristor to a series of pulse stimuli, which can be used to parameterize the model of Du et al. (2015). The oro (Ju and Kim, 2024) provide a detailed potentiation and decay curve for a single, fixed voltage, and current measurements after each pulse for varying voltage pulses. Importantly, the authors provide data for a potentiation-decay experiment for a fixed voltage, and a varying-voltage potentiation experiment. As such, we employed a two-step approach, in which we first obtained the time constant τ from the potentiation and decay curves, and then the other parameters following analysis of the experiments with varying pulse voltages.

For the first step, we recovered the conductance over time *G*(*t*) from Ju and Kim (2024), replicating the trace tenfold to enhance robustness. A pulse-train voltage *V*(*t*) was constructed from the provided methods description by Ju and Kim (2024). To recover τ, simulations were performed using a reduced model (see Equation 5), incorporating backpropagation through time for parameter optimization. This reduced model was constructed from the original model in Equations 1 and 2 by substituting in the read and write voltages and collapsing constant expressions into singular constants. With a fixed write voltage of 4 V, these equations reduce to


(5) $$\dot{G}(t)=-\frac{G(t)-G_{min}}{\tau }+\{\begin{array}{cc} \text{A} & V(t)=4\\ 0 & V(t)=0 \end{array}$$

where *G* is the measured conductance for the fixed 0.7 V read voltage and *A* is a constant. To estimate the unknown parameters *G*_*min*_, *A* and τ, the mean-squared error (MSE) loss of the simulated conductance *G*(*t*) was minimized against the recorded data using the Adam optimizer (Kingma, 2014 via Babuschkin et al., 2020) with a learning rate of 10^−2^ for 10,000 steps, ensuring alignment between the simulated and target behaviors. This led to the tuning result presented in Table 1, which—in the context of the full model—led to a set of constraints for the next optimization round.

**Table 1:** Parameter tuning of the simplified model on the potentiation and decay experiment.

**Parameter**	**Value**	**Unit**	**Constraint**
τ	11.7	ms	τ = 11.7
*A*	1.28	uS/ms	–
*G*_min_	2.18	uS	$\left(1-w_{\text{min}}\right)\alpha \left(1-e^{-0.7\beta }\right)+w_{\text{min}}\gamma \sinh\left(0.7\delta \right)=0.7\cdot 2.18$

These give rise to two constraints in the second tuning step on the full model. The decay time constant τ is used directly in the final model. *G*_min_ does not exist in itself in the full model, yet we can derive a constraint by filling in the 0.7V read voltage used by Ju and Kim (2024) in Equation 1.

For step two of the memristor model definition, we used the measured varying-voltage pulse experiment from Ju and Kim (2024). To further constrain the parameters within biological ranges, we used the list of soft constraints shown in Table 2 for optimization. To prevent overshoot due to the forward-euler discretization scheme, *w* was clipped between *w*_*min*_ and 0.99 in addition to the multiplication of ẇ with the window function. These parameters were encoded in the loss as a weighted sum. The main loss was the mean squared error between the model and data current. The Adam optimizer was used for 1,000,000 steps with a learning rate of 10^−3^. This led to the final parameters as shown in Table 3, Figure 2F.

**Table 2:** Parameter constraints and their weights, as encoded in the loss.

**Weight**	**Expression**	**Rationale**
10^3^	${\left(w_{\text{min}}-0.15\right)}^{2}$	*w* should start low
10^2^	∑*wH*(*w*−1)	*w* should stay below 1
10^4^	|α|·*H*(−α)	parameter can not be negative
10^4^	|β|·*H*(−β)	*sup*.
10^4^	|γ|·*H*(−γ)	*sup*.
10^4^	|*w*_min_|·*H*(−*w*_min_)	*sup*.
10^4^	|λ|·*H*(−λ)	*sup*.
10^4^	|η|·*H*(−η)	*sup*.
1	(β−0.5)^2^	around 0.5 is a right value

*H* is the Heaviside step function.

**Table 3:** Memristor model parameters.

**Parameter**	**NbOx**	**WOx**	**Unit**
τ	11.7	0.05	ms
α	0.0271	0.01	uA
γ	11.138	10	uA
β	0.503	0.5	1/V
η	0.739	8.0	1/V
δ	0.739	4.0	1/V
*w*_min_	0.117	0.1	–
λ	0.0155	0.001	–

NbOx-memristor parameterization is the result of our methods, while WOx-memristor parameters are taken from Du et al. (2017).

### 2.2 Scaling the HH model

**Figure 3:**
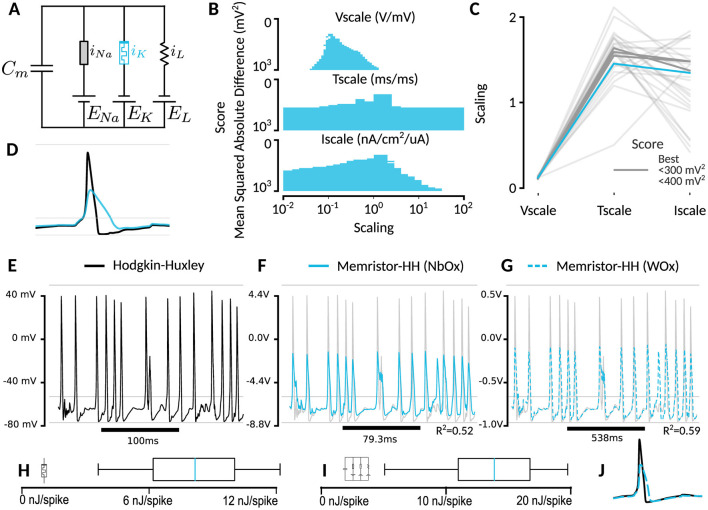
Suitability of NbOx-memristor for emulation of the Hodgkin-Huxley potassium ion-channel. **(A)** Hodgkin-Huxley with potassium ion channel replaced by memristor model. **B)** Design space exploration over required scaling of voltage, time and current. Score is calculated using the mean squared error. **(C)** Correlation between scaling factors for different scores. Best refers to the best found solution in the grid search from **(B)**, while grayscale corresponds to different thresholds. The scaling value corresponds to the different voltage, time and current re-scaling of the passive components. **(D)** Action potential shape comparison. Memristor-emulated Hodgkin-Huxley channel has lower peak amplitude, and slower repolarization phase. **(E)** Desired Hodgkin-Huxley trace. **(F)** Best NbOx-memristor emulated trace with corresponding different voltage and time scales. **(G)** WOx-memristor results. **(H)** Distribution of energy consumption through the memristor in the n = 13 spikes. **(I)** Similar to H, but for the entire HH circuit in the same unit scaling as the memristor. **(J)** Action potential shape comparison as in **(D)**, but against a simulated WOx memristor with 5x lower τ time-constant.

The goal of this study is to find out whether oxygen-vacancy migration memristors are suitable for the emulation of the potassium ion channel in the Hodgkin-Huxley model. Using the parameterized model of the WOx and NbOx memristors, we can now simulate these in a full HH simulation. As such, we performed simulations of the neuron model, in which the potassium channel was replaced by our memristor model (Figure 3A). This, however, corresponds to different scales in voltage and current, i.e., the HH model deals in mV-order voltages, while the memristor responds to V-order voltages. The capacitance of the HH model is expressed in capacitance per membrane area, while real-world capacitors use units of capacitance. Voltage- and time-scaling factors correspond to varying the passive components of the model, i.e. having a different membrane capacitance and leak resistor, but do not otherwise present more difficulties. Current scaling could correspond to either similar passive-device scaling or an increase of the area of the memristor device. In general, the scaling factors should affect the passive components (membrane capacitance *C*_*m*_, reversal potentials *E*_*x*_ and resistor *R*_*L*_) as:


(6) $$\begin{array}{cc} C_{m}^{\prime } & =\frac{T_{\text{scale}}}{V_{\text{scale}}I_{\text{scale}}}C_{m} \end{array}$$

(7) $$\begin{array}{cc} E_{x}^{\prime } & =V_{\text{scale}}E_{x}\;x\in \left\{Na,K,L\right\} \end{array}$$

(8) $$\begin{array}{cc} R_{L}^{\prime } & =V_{\text{scale}}I_{\text{scale}}R_{L} \end{array}$$

Thus, a design-space exploration over potential voltage, time and current scaling factors was performed. Of highest importance were the voltage and time scales: the time constant in the memristor should match that of the potassium ion channel.

### 2.3 Memristor-model tuning to fit HH potassium channel

To adapt the memristor model as a replacement for the HH potassium channel, we employed the following procedure. First, an HH simulation was conducted using noisy input generated with Ornstein-Uhlenbeck (OU) noise ($\theta =0.1/ms,\sigma =0.7,i_{app}=ou^{4}\left(nA\right)$ ), and the resulting trace was saved as the ground truth. Next, the potassium channel was removed from the model and replaced with either the NbOx or WOx memristor model. For both simulations, the Euler-Maruyama method (normal samples originating from a PRNGKey(seed=0) split for the number of timesteps) with a timestep of 0.005 ms was used for integration. Time (*T*_scale_), current (*I*_scale_), and voltage (*V*_scale_) scaling were allowed. Initial conditions *w*(0) = *w*_*min*_ and *V*(0) = −60*mV* were used. These correspond to scaling of the passive components in the circuit. Instead, during optimization, the HH model was simulated using default units, while the memristor model was implemented in the HH model by scaling Equations 1 and 2 with the scaling factors:


(9) $$\dot{w}=\;\frac{W\left(w\right)}{{\text{T}}_{\text{scale}}}\left[\lambda \sinh\left(\eta \cdot {\text{V}}_{\text{scale}}\cdot \left(v-E_{K}\right)\right)-\frac{w-w_{min}}{\tau }\right]$$

(10) $$\begin{array}{l} i_{K}={\text{I}}_{\text{scale}}\cdot [(1-w)\alpha \left(1-e^{-V_{\text{scale}}\cdot \beta V}\right)+w\gamma \sinh(\delta \cdot V_{\text{scale}}\cdot \\ \;\;\left(v-E_{K}\right))] \end{array}$$

A search was performed over *T*_scale_, *I*_scale_ *V*_scale_ ranging from 10^−3^ to 10^3^ to identify optimal parameters via the CMA-ES algorithm by Hansen et al. (2024). A randomized search was performed to understand the effect of varying the scaling parameters. The MSE, after removing the 25 ms initial transient, was calculated for each configuration and the parameter set yielding the minimum MSE was selected. Finally, the *R*^2^ score was computed over a fivefold longer simulation to evaluate the fidelity of the fitted model. This led to the final parameters as shown in Table 4, including CMA-ES seeds.

**Table 4:** Memristor model parameters.

**Parameter**	**NbOx**	**WOx**	**Unit**
Seed	507062	592095	–
V-scale	0.11	0.013	volt/mV
T-scale	1.26	0.186	ms/ms
I-scale	1.91	6.317	uA/(uA/cm^2^)

NbOx-memristor parameterization is the result of our methods, while WOx-memristor parameters are taken from Du et al. (2017). Scaling values found to fit passive components to HH model.

Besides, energy usage was calculated for both the emulated ion-channel and the full-HH circuit by integration over power, while applying the scaling from the tuning methods.


(11) $$\begin{array}{cc} E_{K} & =\int_{t_{1}}^{t_{2}}|V_{K}\left(t\right)||i_{K}\left(t\right)|dt \end{array}$$

(12) $$\begin{array}{cc} E_{HH} & =\int_{t_{1}}^{t_{2}}\underset{x\in \left\{K,Na,L\right\}}{\sum }|V_{x}\left(t\right)||i_{x}\left(t\right)|dt \end{array}$$

### 2.4 Simulation setup

Libraries used include JAX 0.6.0 (Bradbury et al., 2018), optax 0.2.4 (Babuschkin et al., 2020), CMA-ES 4.0.0 (Hansen et al., 2024), scipy 1.15.1 (Virtanen et al., 2020) and numpy 1.26.4 (Harris et al., 2020). Simulations were performed on a workstation with AMD Ryzen Threadripper PRO 3955WX CPU and NVIDIA RTX6000 GPU.

## 3 Results

### 3.1 Equation similarity

To accurately emulate the potassium ion channel as a memristor, the memristor's switching mechanism should, to a large extent, have the same steady-state and transient dynamics as the HH model. When we calculate the steady state of the memristor model from Equation 2, ignoring the windowing function for simplicity, we find that in the steady state; *w* is *w*_*min*_ for *V* = 0 and *w* = *w*_*max*_ for $V\geq \sinh^{-1}\left(w_{max}/\tau \lambda \right)/\eta$. For intermediate voltage values, *w* is monotonically increasing for voltages in between, thus visually resembling the required sigmoidal steady-state of the potassium *n* gate (Figure 1D).

The potassium gate dynamics can be rewritten in the form τ(*v*)ṅ = *n*^∞^(*v*)−*n*, to clarify the steady-state and time-constants for a given voltage. We can, to some approximation, do the same for the memristor model. Depending on the input, around *w*≈*w*_*min*_, one of two time-constants (in square brackets) is dominant:


(13) $$\begin{array}{l} {}[\tau ]\dot{w}=\left(w_{min}+\tau \lambda \sinh\left(\eta V\right)\right)-w\\ \text{ for }\tau \lambda \;\sinh\left(\eta \text{V}\right)<\text{1} \end{array}$$

(14) $$\begin{array}{l} {}[\frac{1}{\tau \lambda \sinh\left(\eta V\right)}]\dot{w}\approx w_{max}-w\;\\ \text{ for }\tau \lambda \sinh\left(\eta \text{V}\right)\gg \text{1} \end{array}$$

Now, by expanding the *i*(*V*) characteristic around *V* = 0 (i.e. *V*_*m*_ = *V*_*K*_) for η*V*≪1 and β*V*≪1, we obtain a linear term in *w* for the conductance, and a persistent Schottky leak conductance αβ:


(15) $$\begin{array}{c} i\left(w,V\right)\approx w\left[\gamma \delta -\alpha \beta \right]V+\alpha \beta V+O\left(V^{2}\right) \end{array}$$

Thus, we find that—beyond being a first-order, volatile, voltage-controlled memristor—the memristor model equations also *show a voltage-dependent sigmoidal steady-state, voltage-varying time-constant and contain a linear conductance-state relation* just as the potassium ion channel would. We also find a non-ideal Schottky leak conductance, which can hopefully be minimized. This makes the oxygen-vacancy diffusion memristor a very suitable candidate for implementing the potassium ion-channel in the HH system.

### 3.2 Memristor-model tuning to fit NbOx

Given that the memristor model itself seems appropriate for potassium-channel emulation, we should now obtain a parametrized model corresponding to the actual WOx and NbOx candidate memristors, such that we can verify the suitability of these memristors in HH simulation. A two-phase approach was used to fit the NbOx-memristor model to the available experimental data (Figures 2A, E). First, a detailed potentiation/decay curve was used to recover the timeconstant τ in the model (Figures 2B, C). Secondly, to recover the constants of the full model (Figure 2D), varying pulse voltages were used (Figures 2E, F). We found that this allowed for too much freedom in the constants, so we forced some of the constants to stay close to reasonable physical ranges (see *Methods* section). For example, a first optimization would lead to a Schottky β of 10 volts, which seemed unnatural. This led to the set of parameters in Table 3. Comparing the results to the known WOx memristor from Du et al. (2015), we found similar values for most model parameters, with the biggest difference observed in η and δ, which became a factor 10x lower for the NbOx-memristor. This corresponds to the smaller difference between potentiation and decay rate observed in NbOx.

### 3.3 HH fit via system simulation

**Figure 4:**
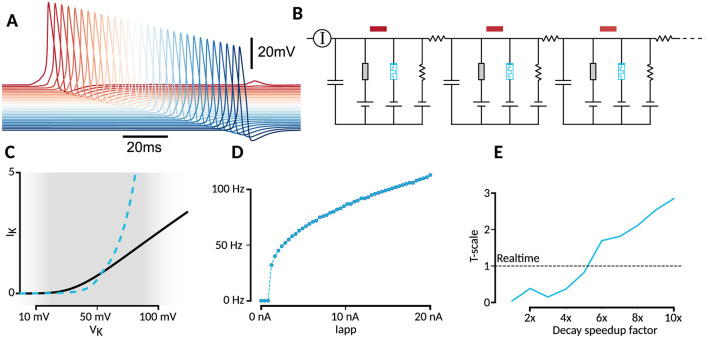
Biological plausibility and integration. **(A)** Simulated axon traces. **(B)** Simulated axon circuit. **(C)** Comparison of steady-states between HH potassium channel (black) and memristor replacement (blue). Approximate operating range of voltages is shown in gray, left side corresponding to the resting potential and right side to the spike height. The sinh tunneling term leads to a large surge in current when the spike get too large, limiting the spike height in the memristor-based HH implementation. **(D)** Frequency-current (F-I) plot shows how increasing current leads to increase in firing rate. **(E)** Artificially decreasing the decay time constants from 50ms by factors between zero and ten, leads to different solutions in the final time-scale solution. For a decrease factor of 5x, correspondings to a 10 ms decay, the WOx memristor should execute the potassium channel dynamics at biological speeds (realtime).

After accounting for the scaling factor, we concluded that the NbOx memristor could be a suitable replacement for the potassium channel, as in our total system simulation the traces turned out to be generally the same (Figures 3E, F). Indeed, the spikes occurred at the same time-points. We found an optimal effective reduction of decay constant τ of ≈26% (Figures 3B, C, F), making the memristor-emulated HH model run an equivalent amount faster than biological time. For voltage, we found that the memristor optimally responds as a potassium ion channel by scaling the cell voltages by a factor of 0.11 volt/mV. This relatively high multiplication factor is due to the very low mV-order biological voltages, which are not high enough to trigger switching in the NbOx-memristor. The WOx memristor exhibited substantially lower performance, operating 5x slower than the biological potassium channel and requiring much lower operating voltages than usual (Figures 3E, G). The role of the potassium ion channel, in biology, is mostly to create the hyperpolarization phase of an action potential, i.e. often reflecting the inhibition of inputs directly after a spike. This is replicated well with the NbOx-memristor model. While the WOx memristor does not align well with the HH model, when reducing the decay time-constant five-fold, it does recreate this hyperpolarization phase quite well (Figures 3I, 4E). The slight 26% increase over real-time is suitable for brain simulation, but might lead to problems in real-time applications, as the simulated model would run faster than biological time.

### 3.4 Relation between spike height and Schottky tunneling

We find that the spikes are now effectively 40% lower (Figure 3D). The extent to which this might be a problem depends on the application at hand. We hypothesized that this is due to the Schottky-tunneling contribution scaling nonlinearly as *sinh* with the bias voltage. This is made more clear when comparing the steady states of the potassium ion-channel and the NbOx memristor model (Figure 4C). To investigate this further, we also simulated a hypothetical memristor where the conductance scales linearly with the internal state variable *w* (Supplementary Figure S1). Indeed, now we obtain both good hyperpolarization as well as similar peak height for the action potentials. However, in certain occasions, we find that this linear memristor model is more susceptible to spikes, leading to a slightly higher spiking rate.

### 3.5 WOx suitability

While the underlying switching mechanisms between the WOx-memristors of Du et al. (2017) and Ju and Kim (2024) seem to be the same, the time-scales at which potentiation and decay happen are different. Firstly, the decay constant τ of Du et al. (2017) is 50 ms, while the decay constant in our model of the NbOx-memristor of Ju and Kim (2024) is 11 ms. At the same time, potentiation is much faster in the WOx-memristor. As such, WOx is a much slower replacement for the potassium ion channel, as can be seen in Figure 3E. However, when we artificially decrease the decay constant to 10 ms, the performance improves beyond that of the NbOx memristor (Figure 3J), and matches biological speeds (Figure 4E). As such it seems like that, while the existing WOx design is not suitable for potassium channel emulation, a fabrication method tuned to the HH-simulation task should perform much better.

### 3.6 Energy usage

Memristors are introduced as an efficient alternative to current digital-based simulation of the HH model. To assess the efficiency of the memristor device, the power through the memristor-emulated potassium channel was integrated during one biological second of simulation. For the NbOx-emulated circuit, an energy expenditure of 0.46 μ*W* through the emulated potassium channel was found, while producing spikes at 51 Hz (Figure 3H). This would correspond to an average of 9.0 *nJ* per spike (including failed initiations). The total energy usage, by including the leak and sodium channels, was estimated to be 0.54 μ*W*, or 11 nJs/spike (Figure 3I). For a very coarse estimate, multiplying this with the 86 billion neurons in the brain would lead to a total power usage of 40 kW for the potassium channel or 46 kW for the entire system.

### 3.7 Circuit emulation and frequency response

The HH equations derive much of their value from the composability to the model, allowing different channels to be inserted or the connection of multiple compartments to model a spatially extended axon. To test the composability of the emulated HH system, we simulated a full 30-compartment axon, as an electrical circuit (Figure 4B). The same OU noise as in the other experiments was applied to the first axonal compartment. Despite the reduced spike amplitude, it remains sufficient to trigger a propagating spike wave in the axon model (Figure 4A), for both NbOx and WOx tuned neurons. Another property of the Hodgkin-Huxley model is the all-or-nothing firing response to varying input current, and an increase in firing rate with increase in applied current. We show the f-I plot in Figure 4D.

## 4 Discussion

### 4.1 Mechanism of memristor operation and model validity

NbOx memristors have shown a wide variety of switching behaviors with putative underlying mechanisms. The stable oxides of NbOx are metal-like NbO and insulators NbO2 and Nb2O5. NbO2-based memristors can be divided into threshold-switching Mott-memristors and analog, volatile, oxygen-vacancy-operated memristors. Driven by current-controlled Joule heating, the crystalline metal-oxide will have a Mott transition at 1081K, creating the typical threshold-switching S-NDR behavior. Below this temperature, certain device configurations will show the voltage-controlled volatile behavior as shown by Ju and Kim (2024). For accurately emulating the HH potassium channel, the device needs to be operated in the latter's regime. Another NbOx device in literature showing such behavior is OKelly et al. (2016), with the decay occurring at timescales in the order of 100's of seconds, underscoring that switching is most likely independent of temperature.

Du et al. (2015) claim that the operation is due to filament growth leading to interfacial modulation of the Schottky barrier. A later investigation by Lin et al. (2023) with in-situ transmission electron microscopy and electron energy loss spectroscopy revealed that the WOx memristor follows a shifting oxygen-vacancy concentration gradient. In both cases, switching is mediated through Schottky-barrier modulation at the interface instead of the bulk material, leading to the same observable electrical behavior. These studies are based on WOx memristors. Ju and Kim (2024) write that the volatile-switching is driven by the same oxygen-vacancy mechanism but they do not provide explicit proof. As can be seen in our results, the model indeed matches the data well. Still, no direct evidence for interface-mediated Schottky modulation is available for volatile NbOx memristors.

We found that the existing WOx memristor model was too slow for real-time brain simulation. To make WOx a more suitable replacement for the HH potassium ion channel, its decay constant needs to be lowered. In general, decay is mediated by diffusion and device geometry. The diffusion coefficient is often expressed as D = D0 exp(Ea/RT). The oxygen diffusion action energy Ea is higher in WO_3_ (1.30 eV, Sikka and Rosa, 1980) than in amorphous Nb_2_O_5_ (1.2 eV, Tsukui et al., 2014) and NbOx (1.17 eV, Hossain et al., 2019), which can partly explain the difference, but the baseline diffusion coefficient D0 is also higher in WOx (0.0683 cm^2^/s) than in NbOx (0.0212 cm^2^/s). Still, this suggests a diffusion coefficient in NbOx that is ≈56x times higher than in WOx. To overcome this limitation, doping could be used to tune oxygen diffusion speed (Kilner, 2000; Pyo et al., 2022; Bae et al., 2024), as well as altered device structure with intermediate layers (Bae et al., 2024; Pan et al., 2024).

Beyond fitting a single device, memristor device-to-device variation presents a major challenge for the integration of memristors in end-user applications (Li et al., 2018). However, variability in biological systems is well-known and—according to many studies—even desirable (White et al., 2000; Faisal, 2012; Waschke et al., 2021). Therefore, any device-to-device and cycle-to-cycle variability among memristors in our study is welcome, assuming that it closely matches (or is at least within the range) of the (scaled) biologically observed variability. The considered memristor devices show very low cycle-to-cycle variability (Du et al., 2017; Ju and Kim, 2024), well within tolerable channel noise in biological systems (White et al., 2000). Similar devices have reported device-to-device coefficient of variation of 22% (Roldán et al., 2023; Park et al., 2024), which is much less than biological neuronal variability (Waschke et al., 2021; Goaillard and Marder, 2021).

### 4.2 Comparison against existing solutions

Multiple attempts exist in the literature to implement the full HH model with the use of memristors. Notable biorealistic implementations are the Mott-insulator-based and the double-relaxation oscillators (Pickett and Williams, 2012; Feali and Ahmadi, 2017; Yi et al., 2018; Yang et al., 2024). These designs operate by triggering the oscillators, which together evoke a spike-like trace. Some notable differences exist to the actual HH model. For example, a non-biological hyperpolarization can be observed before each spike. The circuit does not have a membrane capacitor; instead, capacitors are used to construct the two oscillators. In general, the mechanism of operation, via relaxation-oscillators, is very different from the HH-type voltage-controlled opening and closing of gates. As such, these designs do not present an accurate implementation of biological neurons (Nabil et al., 2022; Landsmeer et al., 2025).

Direct comparison against existing CMOS applications is non-trivial as energy-usage numbers are hardly reported in literature and models vary between experiments. However, for completeness we have some comparison points. On FPGA, a detailed 3-compartmental neuron with 9 ion-channels, without synaptic connections, requires up to 46.8W for 672 neurons. Extrapolating this to the two ion-channels of the HH model gives an estimate of 15mW per realtime neuron (Miedema et al., 2020). The same model on a A100 GPU, but with sparse synaptic connections, requires 103.8W for 729 cells, while running faster than realtime at 0.7s/s, leading to an estimate of 45 mW per realtime neuron (Landsmeer et al., 2024). Energy reported for a 1000-neuron simulation using a general purpose brain simulator range from 11W/neuron on CPU to 6W/neuron on GPU (De Schepper et al., 2022). A very simplified Izhikevich model using a coarse 0.5 ms timestep and integer arithmetic on the SpiNNaker system requires 0.2 mW/neuron (Sharp et al., 2012). These numbers are all orders-of-magnitude larger than estimated in our study at 0.76 μW/neuron. This shows the potential of direct emulation of ion channels using a single device per channel.

### 4.3 Future designs

To emulate the potassium ion-channel in the HH system more accurately, new memristor devices should be designed with this goal in mind, rather than repurposing memristors with different application goals. As shown both theoretically and via simulations, oxygen-vacancy migration is the right underlying mechanism to support both voltage-controlled activation/potentiation and deactivation/decay when no bias is applied. For example, a version of the WOx memristor with reduced decay-time constant performs already much better. By having a linearized conductance response as opposed to the *sinh* dependence, high accuracy can be achieved.

Moving from a pure brain-simulation application to an implantable neuroprostesis, would require focus on tissue compatibility. Current operating voltages are too high for interfacing with real neurons. Various mechanisms have been explored to obtain memristor in operating the biological mV/nA regime (Wang et al., 2023; Fu et al., 2020). How this would transfer to a memristor with the same requirements as the potassium ion channel has not yet been explored. Another option could be translating the voltages at the device/tissue interface.

Efficiency-wise, current memristor technology also seems an order-of-magnitude ahead of current digital solutions. However, the simulated power consumption in this study is still 2 to 3 orders of magnitude higher than that of biological brains. This seems to be largely because of the difference in voltage scales: memristors respond to voltage-order biasing while biology operates at mV-order scales. As such, even more efficient designs could try to leverage this reduction in voltage range.

## 5 Conclusion

Computational neuroscience is dependent on the simulation of ever-growing models of the brain. At the same time, applications of neuroscience models, including neuroprosthesis, soft-robotics and edge-AI, call for bringing these models to embedded formats. This creates a requirement for new brain-simulator platforms that are both efficient and accurate. Memristors, by their theoretical direct mapping as ion channels in the brain, could be a most suitable hardware substrate for said platforms.

This work investigated how oxygen-migration memristors, WOx and NbOx, could function as direct replacements of the potassium ion-channel in the standard Hodgkin-Huxley model. We showed that, with some loss of accuracy, an existing NbOx memristor could be repurposed for that goal, while the WOx memristor, due to a larger ratio between potentiation and decay time-constants, was not as suitable for this purpose. By decreasing the decay constant of the latter five times during simulations, we could approximate the voltage-curves of the HH action potential with more accuracy. Future works should focus on device tuning toward a linearized response, hardware integration, tissue compatibility and finding the sodium memristor replacement.

## Data Availability

Publicly available datasets were analyzed in this study. This code can be found here: https://github.com/llandsmeer/frontiers_nbox_k.
