# Supplementary material for: Efficient implementation of the Hodgkin-Huxley potassium channel via a single volatile memristor
Source: Front Neurosci. 2025 Jul 18;19:1569397. doi: 10.3389/fnins.2025.1569397 (PMC12313636; doi:10.3389/fnins.2025.1569397)
Supplement: Supplementary file 1 [file Data_Sheet_1.pdf]

Supplementary Material

1 SUPPLEMENTARY TABLES AND FIGURES

1.1 Figures

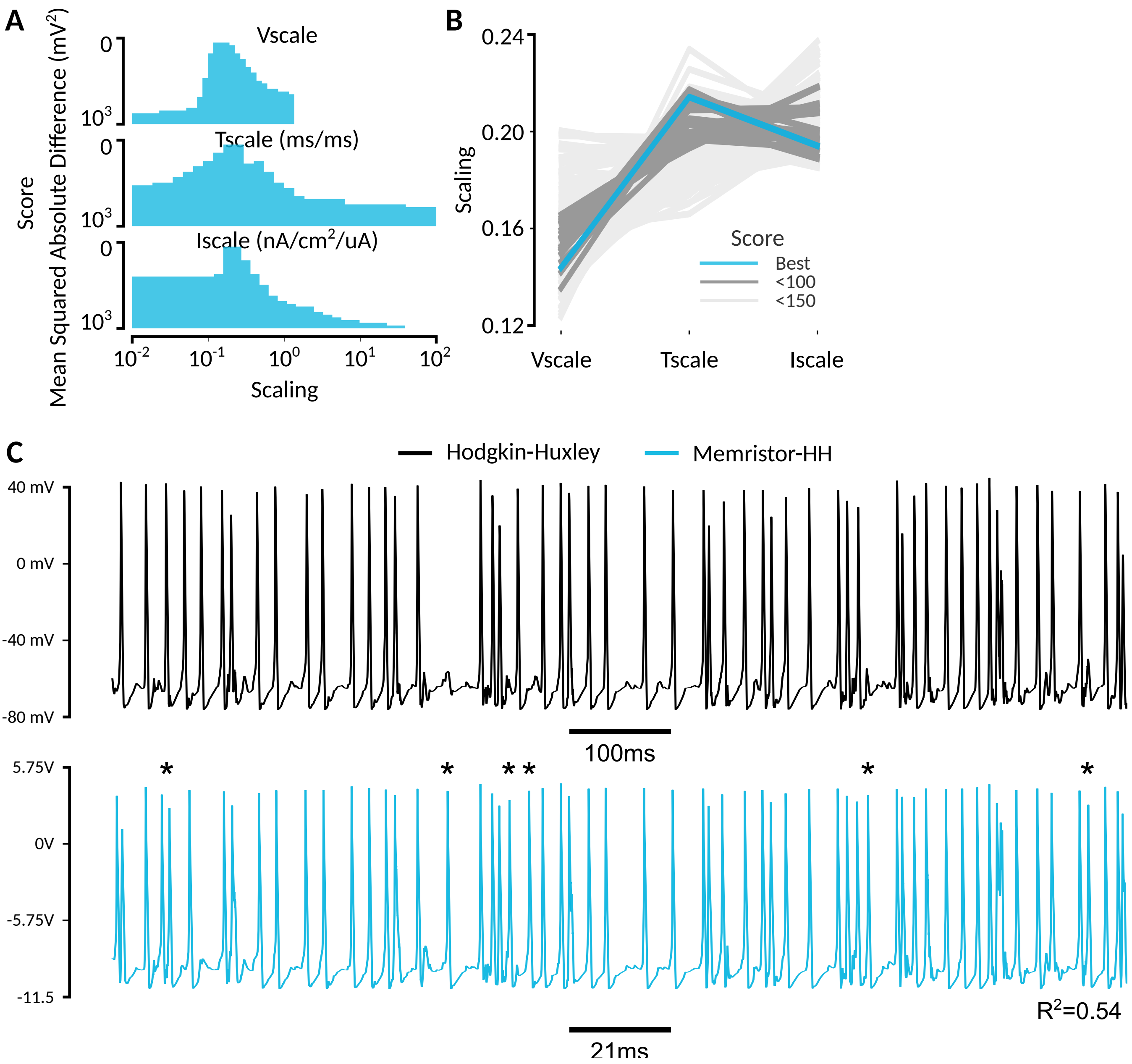

Figure S1. Alternative memristor model, replacing the Schottky barrier with a linear conductance. **A)** Score distributions after sampling scaling factors **B)** Scaling correlation between good results **C)** Resulting voltage trace after, comparing the Hodgkin-Huxley model with the memristor-emulated version
